# Assessing the Clinical Influence of Chronic Total Occlusions (CTOs) Revascularization and the Impact of Vascularization Completeness on Patients with Left Ventricular (LV) Systolic Dysfunction

**DOI:** 10.1155/2022/9128206

**Published:** 2022-08-10

**Authors:** Xi Wu, Jie Cai, Qizhou Zhang, He Huang

**Affiliations:** Xiangtan Central Hospital, Hunan, Xiangtan 411100, China

## Abstract

**Objectives:**

This paper intends to assess the clinical influence of chronic total occlusions (CTOs), revascularization, and the impact of vascularization completeness on patients with left ventricular (LV) systolic dysfunction.

**Background:**

The roles of CTO vascularization in clinical benefits remain conflicting. In addition, data concerning the different results of CTO vascularization and vascularization completeness according to LV systole function were assessed.

**Methods:**

From an overall 918 consecutive patients with at least one CTO, 281 patients with ejection fraction (EF) ≤40% accepted coronary angiographic analysis between Jan 1, 2012, and Dec 31, 2020, and 29 patients were excluded. Subsequently, 261 patients were grouped into the successful CTO-PCI revascularization group (SCR, *n* = 135) and the optimal medical therapy group (OMT, *n* = 126). The prognosis influence of successfully finished CTO-PCI and complete revascularization on survival was evaluated. The primary endpoint was cardiac mortality, and the secondary endpoints were major adverse cardiac and cerebrovascular events (MACCE).

**Results:**

After a median follow-up time of 38.02 months, the cardiac mortality (*p*=0.037) and MACCE (*p*=0.001) were more remarkable in the OMT group than in the SCR group. Moreover, patients with CTO-PCI had survival benefits from complete revascularization for MACCE (*p*=0.025) and cardiac mortality (*p*=0.041). Based on multivariable Cox proportional hazards regression analysis, age ≧ 75 years (HR: 3.443, 95% CI 1.719–6.897, *p* < 0.001) predicted a worse probability of cardiac mortality. Additionally, previous PCI (HR: 1.592, 95% CI 1.034–2.449, *p*=0.035) and previous MI (HR: 1.971, 95% CI 1.258–3.088, *p*=0.003) predicted a worse probability of MACCE, and SCR (HR: 0.499, 95% CI 0.320–0.776, *p*=0.002) was a protection predictor of MACCE.

**Conclusion:**

In patients with LV systole dysfunction (EF ≤ 40%), successfully finished CTO-PCI is related to long-term survival benefits. The benefits were more remarkable in patients with complete coronary revascularization (CCR).

## 1. Introduction

Approximately 15–20% of the entire patients with coronary artery disease (CAD) have coronary artery CTO. With the advancement of intervention treatment apparatus and techniques, percutaneous coronary intervention (PCI) of CTO is becoming more and more popular due to the improved successful rate [1]. Nevertheless, its benefits are still controversial. Certain observation-based research studies [2, 3] contrasting successful versus unsuccessful PCI for CTO have revealed that successfully finished PCI was related to ameliorated results, whereas a few RCTs' discoveries failed to corroborate the benefits of CTO-PCI [4, 5]. On the contrary, left ventricular ejection fraction (LVEF) is one of the most potent prediction factors of cardiovascular events in patients with CAD [6]. It is been revealed that, in patients with ischemia cardiac failure (LVEF < 35%), the existence of CTO was correlated with inferior long-term outcomes [7]. The systematic outcome data of percutaneous complete revascularization of CTO in patients with remarkable LV systole dysfunction are insufficient. This paper intends to clinically assess the prognosis value of CTO complete revascularization, in patients with LV systole dysfunction.

## 2. Materials and Methods

### 2.1. Research Design and Population

The present paper retrospectively studied consecutive patients with at least one CTO and LVEF  ≤  40%, who received coronary angiographic analysis at Xiangtan Central Hospital between January 1, 2012, and December 31, 2020.

The indications for PCI in the presence of CTO were angina, and/or myocardium viability in the territory of CTO. In addition, the willingness of patients to receive PCI therapy was a vital factor for therapy decisions as well. Coronary angiographic analysis was completed by well-trained doctors. The CTO hybrid arithmetic [8] was utilized in every case, beginning with antegrade approaches and in case of failure escalation in the retrograde approach. For bilateral injection, our team utilized 2 access spots, and a combined femoral and radial access was better than double femoral access. Second-generation drug-eluting stents were utilized for every lesion. Patients underwent antiplatelet treatment with aspirin, and patients that received PCI previously or had acute coronary syndrome took an extra P2Y12 suppressor for ≥6 months. The time length of dual anti-platelet treatment was decided by the physician-in-charge. If necessary, patients underwent antianginal and cardiac failure treatment. Normally, patients were subjected to statin treatment. Patients with successfully finished recanalization of the targeted CTO were classified to the “successful CTO revascularization” group (SCR), while patients with unsuccessful PCI and without CTO-PCI were assigned to the “optimal medical therapy” (OMT) group. The database involved demographical, clinical, angiographical, and periprocedural data, as well as in-hospital and partial long-term results.

### 2.2. Definitions of Variables and Clinical Endpoints

CTO was a coronary lesion with thrombolysis in myocardium infarction (TIMI) flow grade 0 in the occluded segment related to a speculated occlusion duration ≥3 months.

The successfully finished CTO-PCI was deemed completed when a TIMI grade 3 anterograde flow was reestablished with a stenosis <30% in the segment [9].

Every patient was confirmed with aberrant systole LV function, diagnosed at the baseline by echocardiography with an EF ≤ 40% [10], and every patient received OMT.

The index date for the analyses of SCR and CTO-PCI failure was the date of the CTO attempt, whereas for the group without CTO treatment, the date was the first coronary angiographic analysis date when CTO was found.

The primary endpoint for SCR and OMT patients' result contrast was cardiac mortality. Secondary endpoints were MACCEs, a combination of all-cause death (ACD), cardiac mortality, relapse of myocardium infarction, targeted lesion angiogenesis, rehospitalisation, cardiac failure, and stroke.

Cardiac mortality was considered mortality within 7 days posterior to MI or stroke, mortality related to cardiovascular intervention, mortality within 1 month posterior to CABG or within 1 week posterior to PCI, or unexpected mortality because of ischemia-related cardiovascular illness, and mortality within 1 day posterior to the occurrence of symptoms with no clinical or postmortem proofs of other causes.

Target lesion revascularization was described as repeated PCI of the treated CTO damage or repeated PCI of non-CTO lesions in the CTO vessel.

Three-vessel coronary stenosis was considered a ≥75% diameter stenosis of the 3 main epicardial coronary arteries or the major branch by evaluation visually, and left main (LM) stenosis was considered LM stenosis ≥50% of the diameter.

The intricacy of CTO was evaluated using the J-CTO scoring [11]; a J-CTO scoring ≥2 was considered an intricate CTO [12].

Syntax scoring was computed for all patients to denote the seriousness of CAD [13].

Complete revascularization was considered the successfully-finished revascularization of the entire lesion (≥75% stenosis) in main epicardial coronary vessels or the major branch (Φ ≥ 2 mm) in the course of the index hospitalisation or at a staged procedure in 1 month posterior to discharge from the index hospitalisation [14].

Dyspnea and angina were evaluated as per NYHA functional class and Canadian Cardiovascular Society class, separately, prior to the indexed CTO procedure.

The entire data were acquired by doctors from novel hospitalisations or by phone calls and/or ambulatory visits completed 6 months posterior to PCI and at least once annually. The patient's general doctor or referring doctors were contacted directly to ensure the accurate death reason if necessary. Every case of heart-related death and abrupt death was reviewed and verified by 2 separate doctors blinded to the angiogenesis information. Every death was deemed as heart-related unless noted otherwise.

### 2.3. Statistics

Baseline features were expressed as average ± SD (or mid-values and first and third IQRs) for continuous variates and were in contrast to Student's *t* test or Mann–Whitney test or Wilcoxon test. Categorical variates were presented as frequencies and proportions (%) and were contrasted by Chi-square or Fisher's extract statistics. The curves of survival were completed using the K-M method and contrasted with the log-rank test. Cox proportion risk approaches were utilized to speculate the roles of several independent variates in the risks of side effects in all the follow-up patients. Every factor that showed significance in univariable analysis (*p* < 0.1) was afterwards tested by multivariable analysis. The outcomes were presented as modified hazard ratios (HRs) with a related 95% CI. Every analysis was 2-tailed and *p* < 0.05 had significance on statistics. Every analysis was completed via SPSS 26.0 (version 26.0, Chicago, IL, USA).

### 2.4. Results

**Figure 1:**
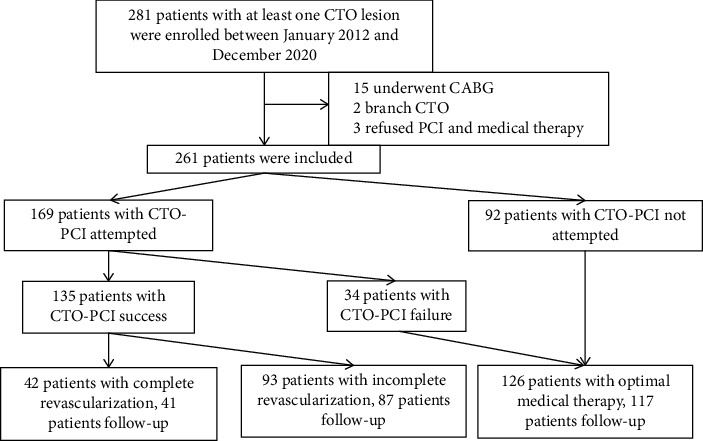
Study flowchart. CTO, chronic total occlusion; PCI, percutaneous coronary intervention; CABG, coronary artery bypass grafting.

An overall of 261 patients with at least 1 CTO and EF ≤ 40% were selected herein (Figure 1). Of those patients, 135 (51.7%) were in the SCR group and 126 (48.3%) were in the OMT group. The OMT group involved patients with original CTO-no PCI (*n* = 92) and unsuccessful CTO-PCI (*n* = 34). The successful rate of PCI was about 78.5% (135 success/172 attempts).

  Baseline characteristics: baseline clinical and angiographical features between the SCR group and the OMT group are summarized in Table 1. Patients receiving SCR treatment were younger (64.75 ± 9.03 vs. 68.41 ± 9.57 years, *p*=0.020), and they had histories of more frequent MI (54.1% vs. 31.7%, *p* < 0.001) and PCI (20.0% vs. 6.3%,*p*=0.002). Conversely, patients receiving OMT treatment had greater J-CTO scoring (2.72 ± 0.76 vs. 2.54 ± 0.94, *p*=0.042) and J-Channel scoring (2.60 ± 0.86 vs. 1.88 ± 1.17, *p* < 0.0001). The symptoms of angina and dyspnea displayed no remarkable diversity in these groups.  Table 2 summarizes the baseline and angiographic features between the complete revascularization group and the incomplete revascularization group. Patients treated with incomplete revascularization were older (66.18 ± 8.69 vs. 61.57 ± 9.03, *p*=0.007), and they had greater J-CTO scoring (2.69 ± 0.92 vs. 2.17 ± 0.88, *p*=0.001), J-Channel scoring (2.34 ± 0.98 vs. 0.69 ± 0.64, *p* < 0.001) and three-vessel stenosis incidence (28.6% vs. 80.6%, *p* < 0.001).
  Clinical follow-up: in contrast to SCR patients, patients with OMT had a remarkably greater rate of MACCE (51.3% vs. 30.5%, *p*=0.001), ACD (23.9% vs. 13.3%, *p*=0.033), cardiac mortality (17.9% vs. 8.6%, *p*=0.037), rehospitalisation (46.2% vs. 16.4%, *p* < 0.001), and cardiac failure(27.4% vs. 10.9%, *p*=0.002) (Table 3). Rehospitalisation happened more often in OMT patients than in SCR patients (46.2% vs. 16.4%, *p* < 0.001). OMT patients suffered more cardiac failure (27.4% vs. 10.9%, *p*=0.002) than SCR patients. Moreover, CTO-PCI patients had survival benefits from complete revascularization for MACCE (17.1% vs. 36.8%, *p*=0.025) and cardiac mortality (0 vs. 12.6%,*p*=0.041).

**Figure 2:**
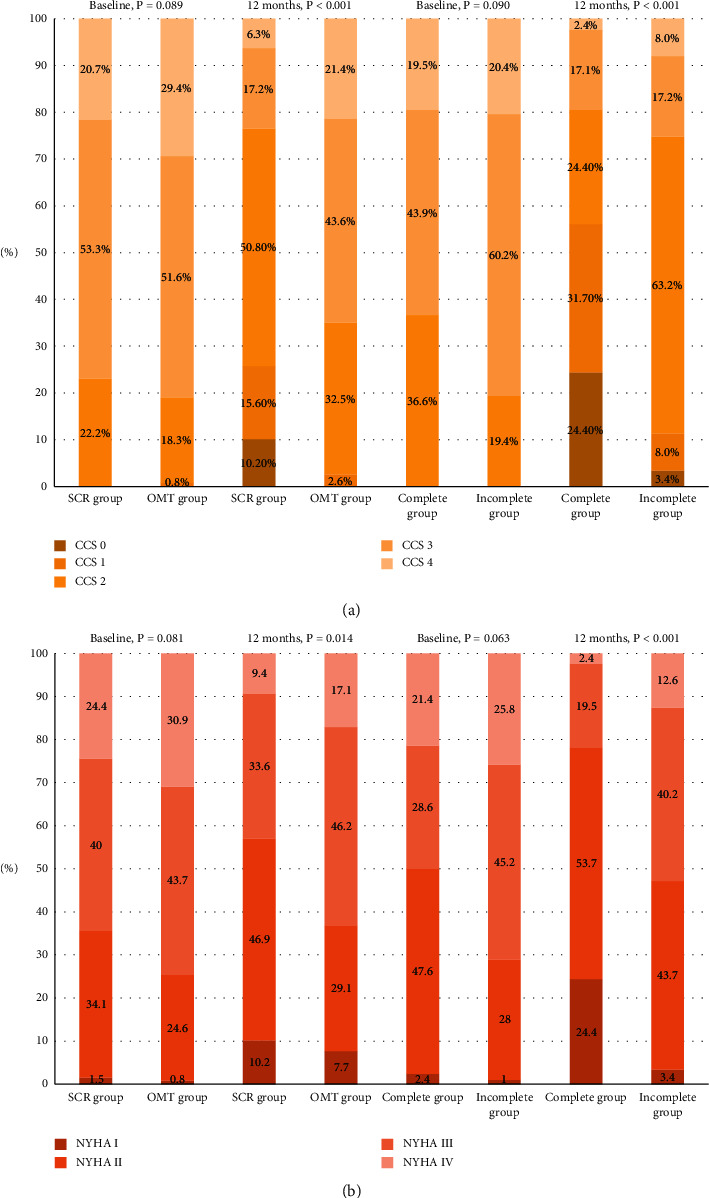
Changes in symptoms. (a) Variations in angina 12 months posterior to CTO-PCI. (b) Variations in breathing difficulty 12 months posterior to CTO-PCI. SCR, successful CTO revascularization; OMT, optimal medical therapy; NYHA, New York heart association; CCS, Canadian cardiovascular society.

Regarding symptoms (Figure 2), dyspnea and angina in SCR patients were significantly improved compared with OMT at the 12^th^ month. Similarly, patients with complete revascularization displayed the improvement of dyspnea and angina compared with incomplete revascularization patients at the 12^th^ month.

**Figure 3:**
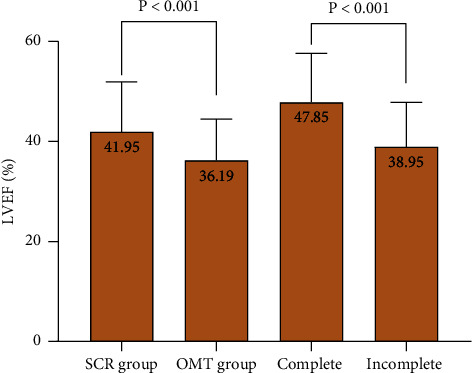
During the 12-month follow-up, the LVEF of SCR patients was significantly increased compared with OMT patients (41.95 ± 9.98 vs. 36.19 ± 8.29, *p* < 0.001), and the LVEF of complete revascularization patients was also significantly increased compared with incomplete revascularization patients (47.85 ± 9.80 vs. 38.95 ± 8.86, *p* < 0.001). Abbreviations: SCR, successful CTO revascularization; OMT, optimal medical therapy.

In survivors, the LVEF of SCR patients was significantly increased compared with OMT patients (41.95 ± 9.98 vs. 36.19 ± 8.29, *p* < 0.001). In the SCR group, the LVEF of complete revascularization patients was also significantly increased compared with incomplete revascularization patients (47.85 ± 9.80 vs. 38.95 ± 8.86, *p* < 0.001) (Figure 3).

**Figure 4:**
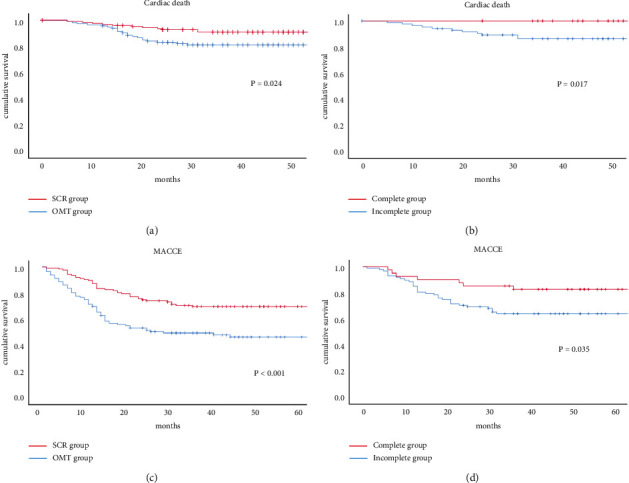
K-M curve of accumulative incidence of (a) cardiac mortality of SCR and OMT patients; (b) cardiac mortality of complete revascularization and incomplete revascularization groups; (c) MACE of SCR and OMT groups; (d) MACE of complete revascularization and incomplete revascularization groups. Abbreviations: SCR, successful CTO revascularization; OMT, optimal medical therapy; MACE, major adverse cardiovascular events.

Kaplan–Meier survival curves estimated cardiac mortality, and MACCE in Figure 4 shows that OMT patients had higher MACCE (*p* < 0.001) and cardiac mortality (*p*=0.024) compared with SCR patients. In the SCR group, incomplete revascularization patients had higher MACCE (*p*=0.035) and cardiac mortality (*p*=0.017) compared with complete revascularization patients.

Based on the entire follow-up, patients with multivariate Cox regressive analyses (Table 4), age ≧ 75 years (HR: 3.443, 95% CI 1.719–6.897, *p* < 0.001) predicted a worse probability of cardiac mortality. Additionally, previous PCI (HR: 1.592, 95% CI 1.034–2.449, *p*=0.035) and previous MI (HR: 1.971, 95% CI 1.258–3.088, *p*=0.003) predicted a worse probability of MACCE, and SCR (HR: 0.499, 95% CI 0.320–0.776, *p*=0.002) was a protection prediction factor of MACCE.

## 3. Discussion

The primary discoveries of the present research are stated below. (a) Successful CTO revascularization was related to long-term survival benefits in CTO patients with LV systole dysfunction (LVEF ≤  40%). (b) Successfully finished CTO revascularization related to CCR offered the greatest clinical benefit. (c) When LVEF was ≤40%, age ≧ 75 years was related to cardiac mortality. Previous PCI and previous MI predicted a worse probability of MACCE, and SCR was a protection prediction factor of MACCE. (d) Successfully finished CTO-PCI in patients with LVEF ≤ 40% was related to a remarkable amelioration of LVEF and symptoms, especially when it came to complete coronary revascularization.

The anticipated beneficial effects from the revascularization of CTO are as follows: (1) better life qualities, (2) better LV systolic functions, (3) long-term survival, (4) elevated tolerance to latent coronary events, and (5) decreased risks of lethal arrhythmia. Patients with severe coronary ischemia causing left ventricular systolic dysfunction are a special group with a high prevalence of ventricular arrhythmia and an elevated risk of sudden death, as well as frequent rehospitalisation with recurrent heart failure and/or angina. They usually have a poor quality of life.

The REVASC trial [5] randomly divided 104 of 205 CTO patients into the non-CTO-PCI group and 101 CTO-PCI group. The study concluded that there were no remarkable diversities in segmental wall thickening of CTO regions between CTO-PCI patients and non-CTO-PCI patients (*p*=0.57), and no statistical significance was observed in LVEF improvement (*p*=0.79). However, after the 12-month follow-up, MACE events in CTO-PCI patients were significantly reduced compared to non-CTO-PCI patients (5.9% vs. 16.3%, *p*=0.02). Flores-Umanzor et al. [15] demonstrated that coronary angiogenesis in contrast to merely medical treatment forecasted lower risks of ACD rates in the elderly with CTO (328 patients), irrespective of angiogenesis methods. Gong et al. [2] divided 563 CTO patients into the non-angiogenesis group (*n* = 263) and successfully finished angiogenesis group (*n* = 300) and revealed that successfully finished CTO-PCI was related to a remarkable amelioration in cardiac mortality and MACCE, and SCR was a protection prediction factor of cardiac mortality (HR: 0.239, 95% CI 0.076–0.751) and MACCE (HR: 0.541, 95% CI 0.353–0.83). Recently, Kook et al. [16] have compared revascularization to OMT for treating CTOs and have discovered that PCI causes improved survival benefits in contrast to OMT irrespective of left ventricular systolic function. Taek Kyu Park et al. [3] enrolled 2024 CTO patients in a single-center registry and followed them for approximately 10 years. They found that CTO-PCI patients had an inferior 10-year rate of cardiac mortality (10.4% vs. 22.3%; HR, 0.44 [95% CI, 0.32–0.59]; *p* < 0.001) than in OMT patients. The comparative decrease in cardiac mortality at 10 years was primarily induced by a comparative decrease between 3 and 10 years (8.3% vs. 16.6%; HR, 0.43 [95% CI, 0.27–0.71]; *p* < 0.001) but not at 3 years (5.7% vs. 5.0%; HR, 1.12 [95% CI, 0.63–2.00];*p*=0.071). A recently finished meta-analysis involving 16 nonrandomised research studies has proved a beneficial effect when it comes to long-term death rate and cardiac mortality in patients undergoing successful CTO recanalization [17]. Another meta-analysis involving eight studies showed that successfully finished CTO-PCI was related to a decrease in long-term death rate (HR: 0.51 95% CI: 0.34, 0.77, *p*=0.01) and MACE (HR: 0.60 95% CI: 0.37, 0.97, *p*=0.04) than in unsuccessful PCI in old people [18].

In our study, for CTO patients with LV systole dysfunction (LVEF ≤ 40%), the improvement of LV systole function, angina and dyspnea symptoms, the incidence of MACE, and cardiac mortality events were better in SCR patients than in OMT patients (*p* < 0.05). Those outcomes reveal that PCI in combination with standard treatment is better than merely medical treatment in left ventricular systolic dysfunction patients with CTO. Diversities were observed in the survival curve of cardiac mortality events and MACCE between the SCR and OMT groups (*p* < 0.05), suggesting that PCI combined with standard treatment displayed survival benefits in patients with left ventricular systolic dysfunction combined with CTO compared to patients with standard medical therapy alone. Certain researchers displayed that the recovery of bloodstream in coronary vessels not only recruited hibernation myocardia to facilitate contraction but also offered vascular access for circulation stem cells [19]. Sufferers receiving successfully finished CTO-PCI with the recovery of anterograde flow and an elevation in distal vascular diameter can benefit from a remarkable decrease in relapse angina and an amelioration in long-term survival compared to patients receiving a failed CTO-PCI [20].

This paper is the first to assess the integrity of revascularization in the presence of CTO in patients with left ventricular systolic dysfunction (LVEF ≤ 40%). Patients with CTO-PCI had survival benefits from complete revascularization for MACCE and cardiac mortality. The improvement of angina, dyspnea, and left ventricular systolic function was better in patients with incomplete revascularization group.

In terms of multiple vessel atherosclerosis illness, survival benefits posterior to successfully finished CTO-PCI could be owing to CCR [21]. Even in patients receiving PCI for several CTOs, the 2-year survival was remarkably improved in patients with full angiogenesis using PCI [22]. In the research of Jang et al. [23], their data revealed that, in CTO patients, acquiring a complete angiogenesis (remaining SYNTAX Scoring = 0) or acceptable incomplete angiogenesis (remaining SYNTAX Scoring = 12) caused remarkably lower risks of cardiac mortality and ACD compared to incomplete angiogenesis (remaining SYNTAX Scoring >12), and those outcomes were consistent with the CABG angiogenesis group with an average follow-up of 42 months. The beneficial prognosis influence of CCR has been substantiated by largescale meta-analysis as well [24]. Hannan et al. revealed that the HR for death rates was the greatest when incomplete angiogenesis was owing to nontreated CTO vessels [25]. Valenti et al., in single-center research involving 460 senior patients, found that the 5-year cardiac survival was remarkably better in the successfully finished CTO-PCI group (84 ± 3% vs. 72 ± 6%;*p*=0.006) and it was ameliorated when CCR was realized (90 ± 3% vs. 68 ± 5%;*p* < 0.001) [26]. Ahn et al. [27] discovered that patients with incomplete angiogenesis by PCI displayed greater risks of ACD compared to patients subjected to CABG treatment. On the contrary, no remarkable diversity was discovered between patients receiving CABG and patients receiving PCI with complete angiogenesis concerning the risks of mortality (aHR: 1.16; 95% CI: 0.83 to 1.63; *p*=0.39) and the combination of mortality, myocardium infarction, and stroke (aHR: 1.14; 95% CI: 0.87 to 1.48; *p*=0.35).

In complex higher-risk and indicated patients (CHIP) [28] with multiple comorbid conditions and remarkably decreased LV functions, the implementation of PCI angiogenesis is deemed quite risky due to technical difficulties and patient features. Kirtaneet al. discovered that the utilization of LV equipment for hemodynamics support in the course of CTO-PCI treatment could be completed with great success rates. Nevertheless, case screening is pivotal, considering the perioperative period complications documented in these patients [29]. Currently, the utilization of the latest angiogenesis method, apart from the technology and procedure aspects, contains the optimum administration of antithrombotic treatment. Patients receiving a CTO-PCI are at greater risk of ischemia and thrombosis. Anti-platelet treatment can be vital for decreasing clinical events [30].

Based on the proofs from randomised trials and registries, we discover that while the actual beneficial effects on prognoses have not been comprehensively revealed, it is evident that the revascularization of CTO induces amelioration in patients' life qualities and decreases angina. Such a result brings deeper questions: which method ought to be utilized in CTO patients with previously unsuccessful angiogenesis or in patients not suitable for CTO-PCI or in patients with remarkable procedural risks?

### 3.1. Research Limitations

However, this paper harbors flaws as follows. (1) Our data originated from a single-center registry. The underlying effects ought to be explored based on more patients, which requires multicentric and randomised trials. This paper was based on assumptions; hence, more prospective research studies are required to explore the advantages of CTO-PCI in LV systole aberrant function. (2) This paper was finished retrospectively and failed to finish the contrast with other treatment methods, such as coronary surgeries. (3) CCR was evaluated merely by anatomical-based definitions. (4) The myocardium viability assay was not implemented in a routine manner, and the latent imbalance of viable myocardia might affect the final outcomes clinically. (5) As patients with eventually unsuccessful CTO-PCI were allocated into the OMT group, these patients might experience poorer outcomes because of the adverse impact of those sufferer subgroups. (6) We failed to evaluate the influence of implanted cardioverter defibrillators on final results clinically. (7) Every procedure was completed by well-trained CTO experts, thereby our outcomes might not be extrapolated to other doctors. (8) No adjustments were completed for multicomparison statistically.

## 4. Conclusions

In conclusion, for patients with LV systolic dysfunction and at least one CTO, successfully finished CTO-PCI was related to long-term survival benefits. The clinical benefits were greater when CCR was realized. The outcomes herein reveal that, in LV systole dysfunction patients, CTO-PCI ought to be taken into consideration to realize CCR. Moreover, age ≧ 75 years predicted a worse probability of cardiac mortality. Previous PCI and previous MI predicted a worse probability of MACCE, and SCR was a protection predictor of MACCE. The findings herein offer new enlightenment for randomised trials required to substantiate our outcomes and can assist physicians in deciding CTO treatment regimens for such high-risk patients.

## Figures and Tables

**Table 1:** Baseline and angiographical features between SCR and OMT patients.

Variable	SCR (*n* = 135)	OMT (*n* = 126)	*p* value
Male gender	105 (77.8)	95 (75.4)	0.663
Age	64.75 ± 9.03	68.41 ± 9.57	0.020
≧75 years of age	22 (16.3)	38 (30.2)	0.008
Hypertension	91 (67.4)	80 (63.5)	0.518
BMI (kg/m^2^)	24.22 (22.27, 26.15)	23.66 (20.89, 25.65)	0.185
Previous smoker	98 (72.6)	84 (87.9)	0.346
Diabetes mellitus	51 (37.8)	37 (42.5)	0.190
Previous MI	73 (54.1)	40 (31.7)	<0.001
Previous PCI	27 (20.0)	8 (6.3)	0.002
Previous CABG	3 (2.2)	0 (0)	0.270
eGFR (ml/min)	69.87 ± 24.60	70.00 ± 25.30	0.966
ICD	4 (3.0)	4 (3.2)	1.000
CPOD	14 (10.4)	19 (15.1)	0.269
Previous stroke	21 (15.6)	16 (12.7)	0.595
Peripheral artery disease	2 (1.5)	0 (0)	0.499
Baseline LVEF (%)	30.92 ± 6.92	31.38 ± 6.65	0.594
Triglyceride (mmol/l and mg/dL)	1.29 (0.97, 1.78)	1.28 (0.94, 1.99)	0.687
Total cholesterol (mmol/l and mg/dL)	4.08 (3.32, 4.95)	3.96 (3.35, 4.93)	0.871
LDL-C (mmol/l and mg/dL)	2.46 (2.07, 2.95)	2.34 (1.95, 3.24)	0.764
HDL-C (mmol/l and mg/dL)	0.98 (0.86, 1.18)	1.04 (0.86, 1.27)	0.215
*Medications*			
Aspirin	131 (97.0)	111 (88.1)	0.007
Clopidogrel/ticagrelor	135 (100)	121 (96.0)	0.059
Statin	130 (96.3)	123 (97.6)	0.795
Beta blocker	131 (97.0)	118 (93.7)	0.243
ACEI or ARB or ARNI	121 (89.6)	120 (95.2)	0.106
Anticoagulatant therapy	84 (62.2)	82 (65.1)	0.700
*Angina (CCS class) p*=0.089			
No angina	0	0	
I	0	1 (0.8)	
II	35 (22.2)	23 (18.3)	
III	72 (53.3)	65 (51.6)	
IV	28 (20.7)	37 (29.4)	
*Dyspnea (NYHA functional class) p*=0.081			
I	2 (1.5)	1 (0.8)	
II	46 (34.1)	31 (24.6)	
III	54 (40.0)	55 (43.7)	
IV	33 (24.4)	39 (30.9)	
*Target CTO artery p*=0.975			
LAD	51 (37.8)	46 (36.5)	
RCA	54 (40.0)	51 (40.5)	
LXC	30 (22.2)	29 (23.0)	
In stent CTO	6 (4.4)	10 (7.9)	0.305
J-CTO scoring	2.54 ± 0.94	2.72 ± 0.76	0.042
J-channel scoring	1.88 ± 1.17	2.60 ± 0.86	<0.001
SYNTAX score	29.32 ± 8.69	30.34 ± 7.51	0.477
*Number of narrowed coronary arteries p*=0.686			
1	14 (10.4)	10 (7.9)	
2	34 (25.2)	29 (23.0)	
3	87 (64.4)	87 (69.0)	
LM disease	13 (9.6)	20 (15.9)	0.14

*Note*. Data are expressed as average ± SD, *n* (%), *n*/*N* (%), or mid-value (interquartile range). Abbreviations: eGFR, estimated glomerular filtration rate; BMI, body mass index; CABG, coronary artery bypass grafting; COPD, chronic obstructive pulmonary disease; ICD, implantable cardioverter defibrillator; PCI, percutaneous coronary intervention; DAPT, dual anti-platelet therapy; ACEI, angiotensin converting enzyme inhibitor; ARB, angiotensin II receptor blocker; CKD, chronic kidney disease; NYHA, New York heart association; CCS, Canadian cardiovascular society; ARNI, Angiotensin receptor enkephalinase inhibitor; J-CTO, Japanese multicenter registry; LAD, left anterior descending; LCX, left circumflflex; RCA, right coronary artery; LM disease, left main disease; LVEF, left ventricular ejection fraction; MI, myocardial infarction; SCR, successful CTO revascularization; OMT, optimal medical therapy; LDL-C, low-density lipoprotein cholesterol; HDL-C, high-density lipoprotein cholesterol.

**Table 2:** Baseline, angiographical, and procedural features between complete revascularization and incomplete revascularization groups.

Variable	Complete revascularization (*n* = 42)	Incomplete revascularization (*n* = 93)	*p* value
Male gender	31 (73.8)	74 (79.6)	0.505
Age	61.57 ± 9.03	66.18 ± 8.69	0.007
≧75 years of age	3 (7.1)	19 (20.4)	0.076
Hypertension	27 (64.3)	64 (68.8)	0.692
BMI (kg/m^2^)	23.76 (21.97, 25.01)	23.76 (21.75, 24.80)	0.714
Previous smoker	28 (66.7)	70 (75.3)	0.306
Diabetes mellitus	12 (28.6)	39 (41.9)	0.180
Previous MI	19 (45.2)	54 (58.1)	0.194
Previous PCI	4 (9.5)	23 (24.7)	0.061
Previous CABG	0 (0)	3 (3.2)	0.552
eGFR (ml/min)	72.00 ± 21.213	67.33 ± 24.614	0.289
ICD	1 (2.4)	3 (3.2)	1.000
CPOD	6 (14.3)	8 (8.6)	0.485
Previous stroke	8 (19.0)	13 (14.0)	0.452
Peripheral artery disease	0 (0)	2 (2.2)	1.000
Baseline LVEF (%)	29.61 ± 6.89	31.39 ± 7.01	0.180
Triglyceride (mmol/l and mg/dL)	1.20 (0.72, 1.62)	1.33 (0.99, 1.90)	0.230
Total cholesterol (mmol/l and mg/dL)	3.92 (3.32, 4.97)	4.11 (3.25, 4.75)	0.641
LDL-C (mmol/l and mg/dL)	2.50 (2.15, 3.24)	2.40 (1.98, 2.93)	0.189
HDL-C (mmol/l and mg/dL)	0.98 (0.86, 1.40)	0.98 (0.84, 1.16)	0.081
*Medications*			
Aspirin	42 (100)	89 (95.7)	0.310
Clopidogrel/ticagrelor	42 (100)	93 (100)	1.000
Statin	39 (92.9)	91 (97.8)	0.353
Beta blocker	42 (100)	89 (95.7)	0.310
ACEI or ARB or ARNI	38 (90.5)	83 (89.2)	1.000
Anticoagulatant therapy	24 (57.1)	60 (64.5)	0.447
*Angina (CCS class) p*=0.090			
No angina	0	0	
I	0	0	
II	15 (36.6)	18 (19.4)	
III	18 (43.9)	56 (60.2)	
IV	8 (19.5)	19 (20.4)	
*Dyspnea (NYHA functional class) p*=0.063			
I	1 (2.4)	1 (1.0)	
II	20 (47.6)	26 (28.0)	
III	12 (28.6)	42 (45.2)	
IV	9 (21.4)	24 (25.8)	
*Target CTO artery p*=0.261			
LAD	20 (47.6)	31 (33.3)	
RCA	15 (35.7)	39 (41.9)	
LCX	7 (16.7)	23 (24.7)	
In stent CTO	2 (4.8)	4 (4.3)	1.000
J-CTO scoring	2.17 ± 0.88	2.69 ± 0.92	0.001
J-channel scoring	0.69 ± 0.64	2.34 ± 0.98	<0.001
SYNTAX score	29.64 ± 2.48	29.25 ± 10.35	0.895
*Number of narrowed coronary arteries p* < 0.001			
1	14 (33.3)	0	
2	16 (38.1)	18 (19.4)	
3	12 (28.6)	75 (80.6)	
LM disease	6 (14.3)	7 (7.5)	0.359
IABP use	5 (11.9)	14 (14.1)	0.791
Retrograde access	8 (19.0)	24 (23.7)	0.513
Number of stents	2.98 ± 0.75	2.57 ± 0.84	0.008

*Note*. Data are expressed as average ± SD, *n* (%), *n*/*N* (%), or mid-value (interquartile range). Abbreviations: eGFR, estimated glomerular filtration rate; BMI, body mass index; CABG, coronary artery bypass grafting; COPD, chronic obstructive pulmonary disease; ICD, implantable cardioverter defibrillator; PCI, percutaneous coronary intervention; DAPT, dual anti-platelet therapy; ACEI, angiotensin converting enzyme inhibitor; ARB, angiotensin II receptor blocker; CKD, chronic kidney disease; NYHA, New York heart association; CCS, Canadian cardiovascular society; ARNI, angiotensin receptor enkephalinase inhibitor; J-CTO, Japanese multicenter registry; LAD, left anterior descending; LCX, left circumflex; RCA, right coronary artery; IABP, intra‐aortic balloon pump; LM disease, left main disease; LVEF, left ventricular ejection fraction; MI, myocardial infarction.

**Table 3:** Contrast of clinical results.

Variable	SCR (*n* = 128)	OMT (*n* = 117)	*p* value

MACCE	39 (30.5)	60 (51.3)	0.001
All-cause death	17 (13.3)	28 (23.9)	0.033
Cardiac mortality	11 (8.6)	21 (17.9)	0.037
Rehospitalisation	21 (16.4)	54 (46.2)	<0.001
Heart failure	14 (10.9)	32 (27.4)	0.002
Target vessel revascularization	2 (1.6)	7 (6.0)	0.134
Recurrent myocardial infarction	4 (3.1)	8 (6.8)	0.239
Stroke	2 (1.6)	4 (3.4)	0.599

Variable	Complete revascularization (*n* = 41)	Incomplete revascularization (*n* = 87)	*p* value

MACCE	7 (17.1)	32 (36.8)	0.025
All-cause death	2 (4.9)	15 (17.2)	0.091
Cardiac mortality	0 (0)	11 (12.6)	0.041
Rehospitalisation	6 (14.6)	15 (17.2)	0.802
Heart failure	2 (4.9)	12 (13.8)	0.228
Target vessel revascularization	1 (2.4)	1 (1.1)	1.000
Recurrent myocardial infarction	1 (2.4)	3 (3.4)	1.000
Stroke	0 (0)	2 (2.3)	0.83

*Note*. Data are expressed as numbers or proportions (%). Abbreviations: MACCE: major adverse cardiac and cerebrovascular events, a combination of ACD, cardiac mortality, relapse myocardium infarction, targeted vascular angiogenesis, rehospitalisation, cardiac failure, and stroke.

**Table 4:** Independent prediction factors of cardiac mortality and MACCE in all follow-up CTO patients.

	HR	95% CI	*p* value
*Cardiac mortality*			
3-vessel disease	1.072	0.309–3.722	0.913
Complete revascularization	0.777	0.299–2.019	0.605
Previous PCI	1.117	0.489–2.551	0.793
Previous MI	1.157	0.546–2.450	0.704
SCR	0.558	0.263–1.184	0.129
≧75 years of age	3.443	1.719–6.897	<0.001

*MACCE*			
3-vessel disease	0.915	0.527–1.590	0.753
Complete revascularization	0.467	0.198–1.100	0.082
Previous PCI	1.592	1.034–2.449	0.035
Previous MI	1.971	1.258–3.088	0.003
SCR	0.499	0.320–0.776	0.002
≧75 years of age	1.343	0.832–2.167	0.227

*Note*. Data are expressed as numbers or proportions (%). Abbreviations. MACCE, major adverse cardiac and cerebrovascular events, a combination of ACD, cardiac mortality, relapse myocardium infarction, targeted vascular angiogenesis, rehospitalisation, cardiac failure, and stroke; PCI, percutaneous coronary intervention; MI, myocardial infarction; SCR, successful CTO revascularization.

## Data Availability

The dataset can be obtained from the corresponding author upon request.

## References

[1] Galassi A. R., Werner G. S., Boukhris M. (2019). Percutaneous recanalisation of chronic total occlusions: 2019 consensus document from the EuroCTO Club. *EuroIntervention*.

[2] Gong X., Zhou Z., Ding X., Li H., Li H. (2021). The impact of successful chronic total occlusion percutaneous coronary intervention on long-term clinical outcomes in real world. *BMC Cardiovascular Disorders*.

[3] Taek Kyu Park, Seung Hun Lee, Ki Hong Choi (2021). Late survival benefit of percutaneous coronary intervention compared with medical therapy in patients with coronary chronic total occlusion: a 10-year follow-up study. *Journal of the American Heart Association.J Am Heart Assoc.*.

[4] Werner G. S., Martin-Yuste V., Hildick-Smith D. (2018). A randomized multicentre trial to compare revascularization with optimal medical therapy for the treatment of chronic total coronary occlusions. *European Heart Journal*.

[5] Mashayekhi K., Nührenberg T. G., Toma A. (2018). A randomized trial to assess regional left ventricular function after stent implantation in chronic total occlusion. *JACC: Cardiovascular Interventions*.

[6] Emond M., Mock M. B., Davis K. B. (1994). Long-term survival of medically treated patients in the coronary artery surgery study (CASS) registry. *Circulation*.

[7] Tajstra M., Pyka P., Gorol J. (2016). Impact of chronic total occlusion of the coronary artery on long-term prognosis in patients with ischemic systolic heart failure. *JACC: Cardiovascular Interventions*.

[8] Brilakis E. S., Grantham J. A., Rinfret S. (2012). A percutaneous treatment algorithm for crossing coronary chronic total occlusions. *JACC: Cardiovascular Interventions*.

[9] Azzalini L., Dautov R., Ojeda S. (2017). Procedural and long-term outcomes of percutaneous coronary intervention for in-stent chronic Total occlusion. *JACC: Cardiovascular Interventions*.

[10] Daneault B., Genereux P., Kirtane A. J. (2013). Comparison of three year outcomes after primary percutaneous coronary intervention in patients with left ventricular ejection fraction <40% versus >/= 40% (from the HORIZONS-AMI trial). *The American Journal of Cardiology*.

[11] Morino Y., Abe M., Morimoto T. (2011). Predicting successful guidewire crossing through chronic total occlusion of native coronary lesions within 30 minutes. *JACC: Cardiovascular Interventions*.

[12] Nagamatsu W., Tsuchikane E., Oikawa Y. (2020). Successful guidewire crossing via collateral channel at retrograde percutaneous coronary intervention for chronic total occlusion: the J-Channel score. *EuroIntervention*.

[13] Serruys P. W., Morice M.-C., Kappetein P. (2009). Percutaneous coronary intervention versus coronary-artery bypass grafting for severe coronary artery disease. *New England Journal of Medicine*.

[14] Hannan E. L., Wu C., Walford G. (2009). Incomplete revascularization in the era of drug-eluting stents. *JACC: Cardiovascular Interventions*.

[15] Flores-Umanzor E. J., Vázquez S., Cepas-Guillen P. (2019). Impact of revascularization versus medical therapy alone for chronic total occlusion management in older patients. *Catheterization and Cardiovascular Interventions*.

[16] Kook H., Yang J. H., Jang J. Y. (2021). Differential clinical impact of chronic total occlusion revascularization based on left ventricular systolic function. *Clinical Research in Cardiology*.

[17] Khan A. A., Khalid M. F., Ayub M. T. (2020). Outcomes of percutaneous coronary intervention versus optimal medical treatment for chronic total occlusion: a comprehensive meta-analysis. *Current Problems in Cardiology*.

[18] Cui C., Sheng Z. (2021). Outcomes of percutaneous coronary intervention for chronic total occlusions in the elderly: a systematic review and meta‐analysis. *Clinical Cardiology*.

[19] Chung C. M., Nakamura S., Tanaka K. (2003). Effect of recanalization of chronic total occlusions on global and regional left ventricular function in patients with or without previous myocardial infarction. *Catheterization and Cardiovascular Interventions*.

[20] Joyal D., Afilalo J., Rinfret S. (2010). Effectiveness of recanalization of chronic total occlusions: a systematic review and meta-analysis. *American Heart Journal*.

[21] Valenti R., Migliorini A., Signorini U. (2008). Impact of complete revascularization with percutaneous coronary intervention on survival in patients with at least one chronic total occlusion. *European Heart Journal*.

[22] Danzi G. B., Valenti R., Migliorini A., Parodi G., Vergara R., Antoniucci D. (2013). Percutaneous coronary intervention for multiple chronic total occlusions. *The American Journal of Cardiology*.

[23] Jang W. J., Yang J. H., Song Y. B. (2017). Clinical implications of residual SYNTAX score after percutaneous coronary intervention in patients with chronic total occlusion and multivessel coronary artery disease: a comparison with coronary artery bypass grafting. *EuroIntervention*.

[24] Nagaraja V., Ooi S. Y., Nolan J. (2016). Impact of incomplete percutaneous revascularization in patients with multivessel coronary artery disease: a systematic review and meta-analysis. *Journal of American Heart Association*.

[25] Hannan E. L., Racz M., Holmes D. R. (2006). Impact of completeness of percutaneous coronary intervention revascularization on long-term outcomes in the stent era. *Circulation*.

[26] Valenti R., Migliorini A., Grazia De Gregorio M. (2019). Impact of complete percutaneous revascularization in elderly patients with chronic total occlusion. *Catheterization and Cardiovascular Interventions*.

[27] Ahn J. M., Park D. W., Lee C. W. (2017). Comparison of stenting versus bypass surgery according to the completeness of revascularization in severe coronary artery disease. *JACC: Cardiovascular Interventions*.

[28] Kirtane A. J., Doshi D., Leon M. B. (2016). Treatment of higher-risk patients with an indication for revascularization. *Circulation*.

[29] Riley R. F., James M., McCabe, Kalra S. (2018). Impella-assisted chronic total occlusion percutaneous coronary interventions: a multicenter retrospective analysis. *Catheterization and Cardiovascular Interventions*.

[30] De Gregorio M. G., Marcucci R., Migliorini A. (2020). Clinical implications of “tailored” antiplatelet therapy in patients with chronic total occlusion. *Journal of American Heart Association*.

